# Exposure of Glass Fiber Reinforced Polymer Composites in Seawater and the Effect on Their Physical Performance

**DOI:** 10.3390/ma12050807

**Published:** 2019-03-08

**Authors:** Matteo Cavasin, Marco Sangermano, Barry Thomson, Stefanos Giannis

**Affiliations:** 1 Dipartimento di Scienza Applicata e Tecnologia (DISAT), Politecnico di Torino, Corso Duca Degli Abruzzi 24, 10129 Torino, Italy; marco.sangermano@polito.it; 2 Element Materials Technology Ltd., Wilbury Way, Hitchin SG4 0TW, UK; barry.thomson@element.com; 3 National Physical Laboratory, Hampton Road, Teddington TW11 0LW, UK; stefanos.giannis@npl.co.uk

**Keywords:** PMCs, GFRPs, seawater exposure, diffusion, ageing, accelerated testing, gravimetric, DMA

## Abstract

An innovative testing methodology to evaluate the effect of long-term exposure to a marine environment on Glass Fiber Reinforced Polymers (GFRPs) has been investigated and is presented in this paper. Up to one-year ageing was performed in seawater, to simulate the environment for offshore oil and gas applications. The performance of an epoxy and epoxy-based GFRP exposed at different temperatures from 25 to 80 °C was quantified. The materials were also aged in dry air, to de-couple the thermal effect from the seawater chemical action. Gravimetric testing and Dynamic Mechanical Analysis (DMA) were conducted in parallel on progressively aged specimens. The effect of specimen geometry and the anisotropic nature of diffusion are comprehensively discussed in this paper. For the quasi-infinite specimens, the results show an exponential increase in the seawater absorption rate with temperature. The methodology allowed for the prediction of the diffusivity at a temperature of 4 °C as 0.23 and 0.05 × 10^−13^ m^2^/s for the epoxy and the epoxy-based composite, respectively. The glass transition temperature reduces as sea water is absorbed, yet the sea water effects appear to be reversible upon drying.

## 1. Introduction

Polymer Matrix Composites (PMCs) are becoming widespread in the oil and gas industry. The potential to exploit their outstanding mechanical properties, along with their reduced density compared to metals, makes them a suitable candidate to overcome the technical limitations of traditional structural alloys for deep-water fossil fuels recovery.

The marine environment is aggressive towards building materials [1,2,3,4]. Both structural steel and concrete are severely attacked by the high saline content of seawater. Standard stainless-steel alloys cannot withstand the corrosive action without other protective means (e.g., passivation techniques, protective paints and liners) [5,6]. Even these approaches can fail to prevent localized corrosion, which will induce catastrophic failures in critical components, particularly if they are not periodically inspected. The oil and gas industry is interested in qualifying materials for eXtreme High Pressure High Temperature (XHPHT) operative conditions [7,8,9], which are usually met in deep-water offshore reservoirs. In these working scenarios, hydrostatic pressure can exceed 700 bar and temperatures can rise above 180 °C. The acidity of the extracted fossil fuels and fluids, which can be rich in CO_2_ and H_2_S, particularly for enhanced oil recovery, poses a further challenge to the material durability [10,11].

Duplex stainless alloys have been introduced for particularly demanding applications in corrosive environments, but their cost rarely makes them a viable option for large structures, such as pipelines in the petrochemical industry [10]. Polymers demonstrate a remarkable chemical inertness and they do not suffer from electrochemical corrosion due to their dielectric nature. However, this is not their only benefit: they are significantly lighter compared to metals. Considering their application in a submerged structure, on average, their relative weight is seven-fold lighter compared to the more common steel alloys. This means a significant reduction in the tensile stresses generated by the weight of pillars or pipelines with a very long vertical drop [8,11]. However, they cannot perform as well as structural materials, as they do not possess the required mechanical strength, in particular, with regards to tensile and creep stress. They are much more effective when combined with engineered fibers whose mechanical properties are far superior, resulting in a fiber reinforced Polymer Matrix Composite (PMC). In this form, not only are they excellent structural materials, but their very low relative density (about 2:1 compared to water) allows designers to exploit the hydrostatic pressure to relieve most of the tensile stress due to the weight of a high drop construction [11]. This is a particularly sensitive design constraint for components such as offshore risers, which are used to transfer the fossil fuel and working fluids from the wellhead, sitting at the seabed, to the surface production and storage facilities. Due to the exhaustion of many onshore and shallow water oilfields, the need to drill at greater depths is pushing the design of these structures to the technical limits of traditional building materials. There is an increasing need for composite and hybrid materials able to withstand XHPHT conditions and to enable the exploitation of deep-water reservoirs [1,11].

There is not yet extensive experience with PMCs when it comes to marine applications. The qualification of composite materials for harsh oil and gas services is less advanced than in the aerospace industry, where specific grades and testing routines have been developed over many decades [12]. Pipelines and petrochemical component manufacturers are finally ready to shift to fully composite structures [8]. The main ongoing issue is that the operative working life for those components should be in the order of tens of years in order for them to be economically viable. Due to the very inaccessible environment in which they are installed, no maintenance should be needed for the entire planned lifespan, lest to incur relevant additional costs, and their durability has to be consistently proven.

To introduce a new material grade in a commercial product, such as a flexible riser, the material has to be fully qualified, first at the specimen level and then as a component prototype, to certify that it is going to be safe and reliable for the whole working life. For long-term performance, testing cannot be carried out at a real timescale, as the technology would be obsolete by the time the product was ready to be introduced to the market. Therefore, there is a call for improved accelerated testing methodologies, which shorten the test length to reasonable timeframes, in order to successfully predict the evolution of the material properties over a few decades.

Many operational parameters can affect the results of these tests, so controlling or excluding the highest possible number will return a more robust methodology. Some of the variables are also interacting in a highly nonlinear way, such as the temperature, diffusivity coefficient, and chemical reactions rate. Even when there are theoretical models available to describe these interactions, they still rely on experimental data to be fit to. Hence, there is a need to map the material properties for certain conditions and their evolution with time. The more representative the simulating environment is of the operative scenario, the more accurate the data that is returned. The understanding of the diffusion kinetics at different temperatures provides useful information for foreseeing how the material will behave when exposed to the operative conditions. Ideally, if the data available is extensive enough, it can be possible to define a time-temperature superposition relation, which will allow data from short-term high temperature testing to be extrapolated to predict long-term performance at lower temperatures. Holding this information, the materials, and component designers can result in making an informed decision in a conservative manner.

In this paper, we investigated suitable approaches to measure and quantify the diffusion of seawater in epoxy-based composites, and attempted to use the glass transition temperature as a means of quantifying the physical and chemical ageing of the materials. To achieve that, exposures at a range of temperatures have been conducted and the effect of the coupon size in obtaining a reliable measure of the diffusivity is discussed.

## 2. Materials and Methods

### 2.1. Materials

The materials used in the experimental work are all commercially available. They were selected as representative for use in a marine environment involving exposure to seawater and it should be noted that the present research work does not aim to formally evaluate the performance of these commercial products.

A two-component Ampreg 26 epoxy resin with amine hardener commercial grade thermoset epoxy was selected. This material is suitable for vacuum assisted infusion and was obtained from Gurit (Newport, UK). The epoxy resin consists of a blend of bisphenol A, bisphenol F, and 1,6 hexanedioldiglycidylether, while the hardener is a blend of amines (polyoxyalkyleneamine, 2,2′-dimethyl-4,4′methylenebis (cyclohexylamine), 4,4′-methylenebis (cyclohexylamine), and 2,2′-iminodiethylamine) [13].

The stitched unidirectional glass fiber fabric (1200 g/m^2^, from Gamma Tensor, Alcoi, Spain) comprises 3B Advantex^®^ SE 2020 Direct Roving made of boron free E-CR glass, which is designed for the production of non-crimped fabrics and has a proprietary sizing specifically designed for excellent adhesion with epoxy resin systems.

Neat epoxy plates were manufactured by compression molding to reduce the formation of voids. A hydraulic press (Mackey Bowley, now Wessex Hydraulic Services, Trowbridge, UK) was used to apply a pressure of about 2 MPa at incremental steps to the viscous resin, hence ejecting residual air from the mould cavity. A rubber seal placed between the mold plates allowed the plates to be adjusted and let the air evacuate. The compression mold also enabled us to control the plate’s planarity. The resin was left to cure for 24 h at room temperature, followed by a post-cure of 5 h at 80 °C. Once the curing was completed, the solidified plate was carefully removed from the mold. The plates were machined by a CNC milling machine to obtain the specimens at the required geometries.

The Glass Fiber Reinforced Polymer (GFRP) composite was manufactured by infusing the unidirectional glass fiber fabric with the same Ampreg 26 epoxy. A vacuum bag infusion process was used. After 24 h curing at room temperature, the GFRP plate was removed from the bag and placed in a forced convection oven (Genlab Ltd., Widnes, UK) at 80 °C to post-cure for 5 h. Two machining methods were used to section the GFRP plates and obtain the required specimens, namely diamond blade cutting (for initial cut) and waterjet (for finishing of the coupons). There was not any effect of the cutting method on the specimens obtained for this test program.

### 2.2. Methods

The evolution of the material properties was monitored by different techniques while the specimens were exposed in simulated working environments, being soaked in substitute seawater. The coupons were immersed in plastic containers and kept at different exposure temperatures (25, 55, and 80 °C) in forced convection ovens. At scheduled intervals, gravimetric measurements were performed following the ASTM D5229M [14] standard procedure, allowing the evaluation of fluid absorption progress. In the first stages, the fluid diffusion proceeded at a higher rate, requiring more frequent sampling, particularly at the higher temperatures, even at just a few hours, to effectively capture the absorption through the weight increase. After saturation, the sampling intervals were prolonged up to weeks. In parallel, Dynamic Mechanical Analysis (DMA) runs were performed to evaluate the glass transition temperature (*Tg*).

#### 2.2.1. Conditioning in Simulated Environments

The gravimetric and DMA test specimens were exposed to substitute (or synthetic) seawater, described by the ASTM D1141 [15] standard procedure and purchased by ReAgent, (Runcorn, UK), in order to simulate the marine environment. The composition of the seawater is presented in Table 1.

The seawater containers where placed in forced convection ovens to reach the equilibrium temperature. Different temperatures were selected to accelerate the diffusion and ageing processes. The selected temperatures were 25, 55, and 80 °C, as it was decided not to exceed this higher temperature, which was already very close to the glass transition temperature of the pristine epoxy. The oven’s temperature was constantly recorded by K-type thermocouples, logging to the lab server.

To evaluate the possible scale and edge effect due to the nominal coupons’ dimensions, five different geometries were chosen and are listed in Table 2.

The specimens, following an initial pre-conditioning to eliminate any possible moisture content (14 days at 50 °C), were introduced in the seawater: this moment is considered the beginning of the conditioning/ageing process. At regular times, the gravimetric specimens were extracted from the containers and were weighed, to monitor the progress of the seawater diffusion, and reintroduced in the simulated environment, in an effort to reduce the exposure to the external atmosphere (5 min at most out of the seawater) to a minimum. Two scales with a 0.1 mg precision were used for weighing the coupons. Complete absorption curves were generated in this way. The results were recorded to monitor the evolution of material properties. At the scheduled end of the conditioning, after about one-year exposure, the remaining specimens were removed from the exposure and either tested “as-is” or re-dried in an oven set at 50 °C, to evaluate the desorption curves and explore any recovery of the elastic properties. As a reference control, some coupons were exposed at the same temperatures, but kept in dry air within sealed tin cans (silica bags were introduced in order to keep the moisture content at a minimum), in order to compare the effect of the temperature ageing only on the materials.

#### 2.2.2. Dynamic Mechanical Analysis (DMA)

Dynamic Mechanical Analysis (DMA) was performed using an RSA 3 analyzer, by TA Instruments (New Castle, DE, USA), to evaluate the glass transition temperature (*Tg*) of the materials. The sampling times were scheduled so as to capture the *Tg* shift in the critical moments of the absorption process. The loading mode was sinusoidal three-point bending. The loading span was of 25 mm. The sample geometry was 2 × 10 × 33 (*h* × *w* × *l***)** and was obtained by cutting part of the respective δ coupons for the neat Ampreg 26 and ε coupons for the GFRP. The temperature ramp was set from 25 up to 150 °C, at a 5 K/min heating rate. Such a higher heating rate was selected (instead of the standard 3 K/min) in an attempt to shorten the period during which the sample was outside the exposure environment. A trade-off between the measurement precision and the accuracy of the “wet” *Tg* measured was required to accelerate the test. The *Tg* transitions were evaluated both by the storage modulus (E’) drop onset and by the tanδ peak. The DMA specimens removed at selected intervals of conditioning were investigated “as-is” and after re-drying in an oven at 50 °C for up to two weeks. In this way, it was possible to evaluate the reversibility of the fluid diffusion on the viscoelastic properties of the materials.

## 3. Results

The gravimetric measurements are reported as a function of the length of the exposure to the simulated marine environment (i.e., substitute seawater). The timescale is expressed as the square root of time, using days as the unit of measure for ease of understanding and to present a consistent representation between the gravimetric measurements and the rest of the tests. The Fickian theory of diffusion states that the mass uptake (i.e., moisture content) is linear to the square root time for the initial part of the $M_{t}$ vs. $\sqrt{t}$ plot. This representation also helps to expand the early stages, when the absorption process is at its highest rate and can be expected to have the highest degree of influence on the material properties. The single data point is an average of the weight of the set of specimens (three replicas for each exposure condition specimen type) involved in a single measurement.

### 3.1. Gravimetric Measurements on Neat Ampreg 26 Epoxy

**Figure 1:**
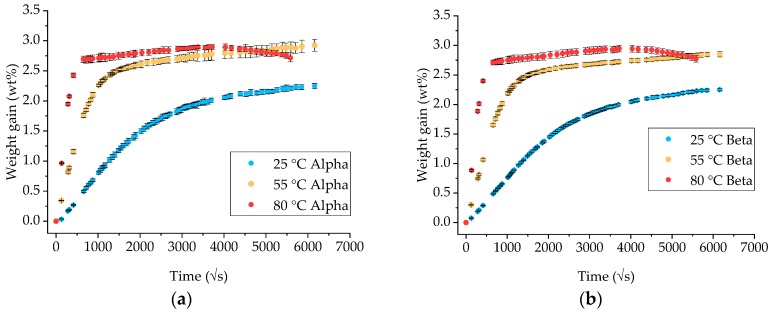

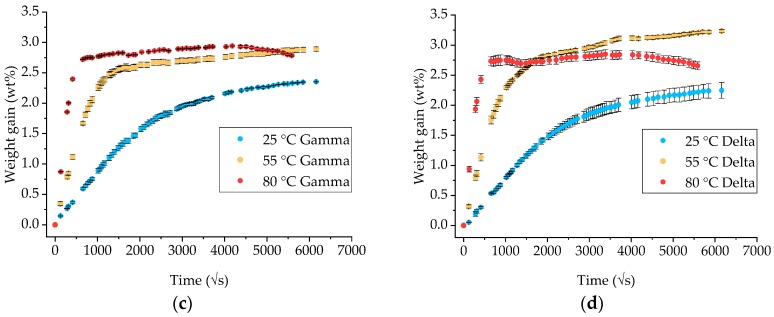
Gravimetric measurements on neat Ampreg 26 coupons of different shape factors: (**a**) Alpha; (**b**) beta; (**c**) gamma and (**d**) delta. (Note: coupons’ dimensions are listed in Table 2). The x-axes are measured in the square root of seconds.

Gravimetric measurements on neat epoxy specimens of different geometries were performed to assess the fluid absorption process and evaluate if any shape factor influences the process. The results are presented in Figure 1 as an average of the measurements of all the specimens available.

The results obtained show that the diffusion process is not completely Fickian. After an initial linear uptake (in $\sqrt{t}$ space), the material reaches a so-called pseudo-saturation [2,16]. A saturation time can be calculated, related to the exposure temperature. However, if the gravimetric measurements are continued, it is evident that the material continues to absorb seawater at a much slower rate, entering another linear regime. It does eventually reach a definitive saturation stage, but at a much later stage. For the 80 °C exposure, after about six months, the graph shows a noticeable decrease in weight, perhaps an indication of chemical degradation.

The ASTM D570 and ASTM D5229 standards describe in detail the design and the methodology of gravimetric testing to evaluate the diffusivity properties for plastics and polymer composite materials. A pivotal feature is the saturation criteria used, which should be a numerical one, and it relies on the convergence of the weight of the coupons as those approach a steady saturated state. Hence, from consecutive measurements, when the relative change in per cent weight gain decreases below a certain threshold, the material can be deemed as in equilibrium. The ASTM D570 standard states that the material is saturated when its weight changes less than 5 wt% or less than 5 mg over three consecutive measures, while the ASTM D5229 (see Equation (1)) is more stringent, and requires a change in weight by less than 0.020 wt% over each of two consecutive reference time period spans, and examination of the weight gain versus (time)^1/2^ plot. The second criterion is much stricter due to composites being less prone to absorbing fluid as inorganic fibers are considered inert.

(1) $$\left|\frac{W_{n}-W_{n-1}}{W_{0}}\right|\times 100<0.020\%\;\mathrm{for}\;2\;\mathrm{consecutive}\;\mathrm{reference}\;\mathrm{time}\;\mathrm{span}$$

This approach has its limitations. In particular, it does not consider the actual saturation level of the material. Therefore, polymers with a very low saturation level or with very low diffusivity can be evaluated as saturated too early, while others more prone to absorbing or showing secondary stages could eventually never reach proper saturation. From experimental practice, it is evident that these criteria are not good enough to capture the time to saturation, but they highly rely on the subjective evaluation of the operator.

In this work, a slightly different saturation criterion is proposed, by evaluating the partial increase over consecutive measurements and dividing this by the overall weight gain to that point. In this way, small changes are more relevant to low diffusing materials compared to more permeable ones. This allows a more robust numerical saturation criterion to be defined:

(2) $$\left|\frac{W_{n}\;-\;W_{n-1}}{W_{n}\;-\;W_{0}}\right|<\left[thresold\;value\right]\;\mathrm{for}\;\left[n\right]\;\mathrm{consecutive}\;\mathrm{reference}\;\mathrm{time}\;\mathrm{spans}$$

Even this criterion is susceptible to the selection of appropriate threshold values. From our analysis, it was found that a value of 0.02 (in other words, less than 1/50 of the overall weight gain) allows the saturation point to be individuated with a good consistency, even when the fluid uptake is very slow, i.e., ambient temperature conditions.

In order to improve the reliability of a numerical criterion for the localization/target of the effective equilibrium, the results obtained using different criteria are compared in Table A1 (see Appendix A).

From the values reported, it can be highlighted that the criterion prescribed by the ASTM D5229M is less precise and reliable, with a large scatter in the values obtained for each specimen geometry at any temperature. The proposed method appears to be more consistent, yet it is susceptible to noisy measurement and still requires a comparison of the $M_{t}$ vs. $\sqrt{t}$ plot. Of the two different threshold values proposed, the 0.02 is preferred as it captures the effective equilibrium at 25 °C in a more accurate way. For these reasons, for the rest of this study, we refer to the effective equilibrium as defined by the residual increment Criterion A.

The values in Table 3 are essential for the evaluation of the diffusivity of the material, as is the consideration of the contribution of the exposed edges of the specimens, hence the deviation from one-dimensional diffusion.

### 3.2. Gravimetric Measurements on GFRP Composite

**Figure 2:**
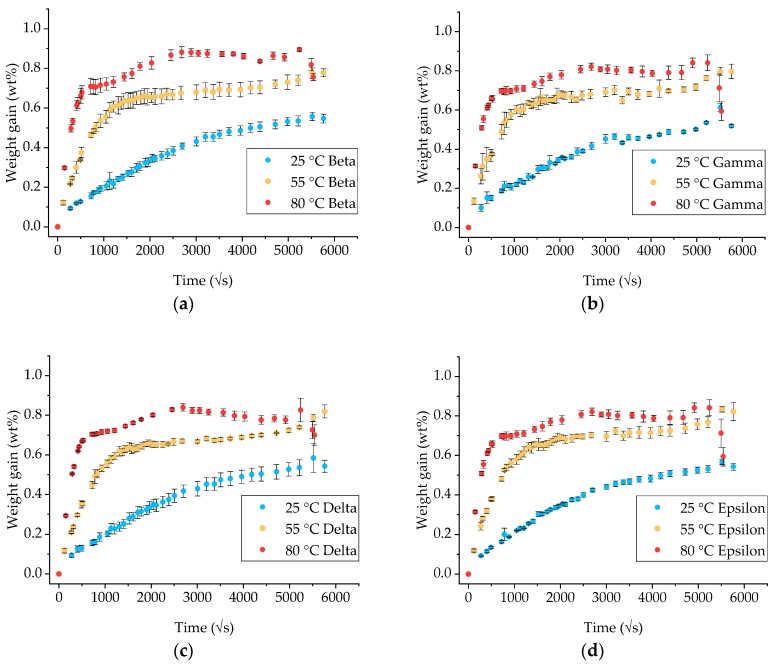
Gravimetric measurements on GFRP coupons of different shape factors: (**a**) Beta; (**b**) gamma; (**c**) delta and (**d**) epsilon. (Note: coupons’ dimensions are listed in Table 2). The x-axes are measured in the square root of seconds.

Similar to the neat epoxy, gravimetric measurements on GFRP specimens of different geometries were performed to assess the fluid diffusion and evaluate the shape factor effects. The results are presented in Figure 2. In this case, the alpha coupons were not used due to their small mass (even considering the relevant inert fiber content), so the change in weight due to the absorbed moisture would be subject to significant experimental error. Instead, coupons coded as epsilon were introduced. These have the same bar geometry as coupons denoted as delta, but the fibers are aligned along the shorter side. The aim is to highlight if the there is any anisotropic effect in the fluid diffusion due to the fiber presence, such as preferential paths or different contributions of the matrix-fiber interphase.

The diffusion at 25 °C appears to be Fickian for the duration of the exposure as was at 55 °C for approximately 250 days when a sudden increase in weight was observed. On the contrary, when exposed at 80 °C, diffusion follows a Fickian curve for about 20 days and then significantly deviates, signifying the beginning of a secondary diffusion stage [17,18].

The moisture uptake at effective equilibrium and the respective time to reach that level were calculated using the residual increment Criterion A (see Equation (2)) with a threshold value of 0.02 and *n* = 2. The results are listed in Table 4. The scatter of the individual measurements negatively affected the precision of determining a consistent point of effective equilibrium, with a large spread between the different specimen geometries, particularly for the exposure at 80 °C. For this temperature, if the threshold value is increased to 0.04–0.05, taking into account the higher degree of noise in the measurements, it is possible to obtain much more accurate saturation times.

For the higher exposure temperatures (55 and 80 °C), a higher amount of seawater appears to be absorbed by specimen type epsilon compared to delta, which could be an indication of an increasing role of the fiber-matrix interphase as the fibers in epsilon are aligned along the shorter side and hence more fiber edges are exposed to the environment. However, the differences are relatively small and no solid conclusion can be derived.

### 3.3. Mechanical Performance

The mechanical performance plays a significant role when selecting a polymer composite material for a structural application in a harsh environment. Amongst the different characterisation techniques, the tensile test is commonly used to evaluate the mechanical performance and measure the Young’s modulus and strength [4,12]. In our study, alongside the gravimetric coupons, tensile test specimens were exposed in seawater over prolonged periods of time. Neat, epoxy specimens were tested to evaluate the degradation in performance of the matrix alone. Likewise, GFRP specimens with unidirectional reinforcement in either the longitudinal or transverse direction were tested to monitor how the fiber- and matrix-dominated mechanical response changes over time. All specimens were tested at different sampling times, chosen in relation to the advancement of the diffusion stages at the exposure temperature, in an attempt to capture the most relevant transitions (i.e., pristine material, linear uptake, Fickian saturation, aged). Although the experimental results of this expensive test program are not presented here, a close correlation between the performance loss, the degree of saturation degree, and the exposure temperature was found and will be comprehensively discussed in a future paper.

## 4. Discussion

### 4.1. Diffusivity Calculation for Neat Ampreg 26 Epoxy

**Figure 3:**
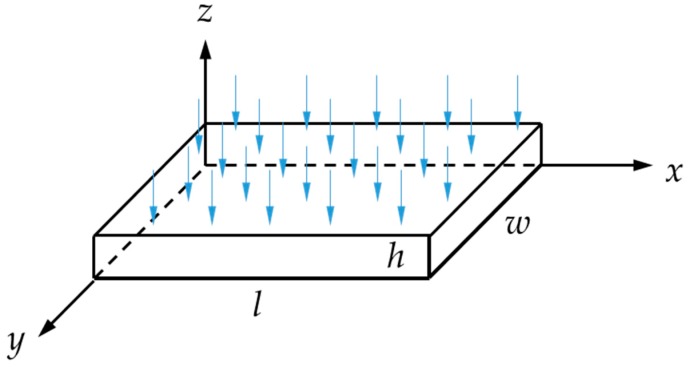
Schematic of rectanguloid isotropic material specimen.

The moisture content in the isotropic (*D*_x_ = *D*_y_ = *D*_z_ = *D*) rectanguloid of Figure 3 is given by the following integral solution to Fick’s equation for one-dimensional diffusion:

(3) $$\frac{M_{t}}{M_{\infty }}=G_{1D}=1-\frac{8}{\pi^{2}}\sum_{j=0}^{\infty }{\left(2j+1\right)}^{-2}\exp\left[-\frac{{\left(2j+1\right)}^{2}\pi^{2}Dt}{h^{2}}\right]$$

To calculate the diffusivity coefficient, *D*, a simplified version (valid as $M_{t}/M_{\infty }\;<\;0.6$) of the above equation was considered:

(4) MtM∞=G1D=4hDπ×t

where, $M_{t}$ is the moisture content at time *t*, $M_{\infty }$ is the moisture content at the Fickian saturation (effective equilibrium), and *h* is the thickness of the specimen. The slope of the $M_{t}$ vs. $\sqrt{t}$ for $M_{t}\;<\;0.6\;M_{\infty }$ (linear diffusion) is equal to

(5) Slope=4M∞hDπ

The above equations do not take into account any diffusion happening from the free edges of the specimens. For that, the three-dimensional problem of diffusion in a rectanguloid needs to be considered and solved. Apart from being very computationally expensive, this has the additional drawback that the diffusivity is not easily determined from the linear initial slot of the $M_{t}$ vs. $\sqrt{t}$ graph. For this reason, the introduction of a correction factor can be considered, as follows:

(6) $$G_{3D}=fG_{1D}$$

and hence,

(7) $$D_{corr}=f^{-2}D_{eff}$$

where, *D_corr_* is the corrected diffusivity coefficient for diffusion through the free edges and *D_eff_* is the diffusivity coefficient measured from the gravimetric experiments.

Shen and Springer [19] and Starink, Starink and Chambers [20] have derived correction factors that are used in this paper to correct the diffusivity coefficients:

Shen and Springer [19]:

(8) $$f_{S\&S}=1+\frac{h}{w}+\frac{h}{l}$$

Starink, Starink and Chambers [20]:

(9) $$f_{SSC}=1+0.54\frac{h}{w}+0.54\frac{h}{l}+0.33\frac{h^{2}}{wl}$$

The results for the neat Ampreg 26 epoxy are presented in Table 5 for the various temperatures and specimen shapes.

For the quasi-infinite coupon size (gamma), the two correction factors converge (only 2% difference) to 1 and therefore, the corrected diffusivities are in better agreement and close to the measured ones from the experimental curves. For more irregular coupon sizes and especially for the very small coupon, the correction factors diverge from 1 and from each other, showing a large range of diffusion coefficients. If all results are compared to the quasi-infinite coupons, then *f*_S&S_ overcorrects the diffusivity values; hence, the *f*_SSC_ is considered more accurate and applicable. This is in agreement with the results presented in [20,21].

The present analysis demonstrates that quasi-infinite coupons are necessary to obtain accurate diffusivity values and, in the case that these are not available due to manufacturing issues (i.e., extracting coupons from a small slab of material), the *f*_SSC_ correction factor should definitely be considered to avoid errors, even up to 30%.

### 4.2. Diffusivity Calculation for the GFRP Composite

As with the isotropic case of the neat Ampreg 26, correction for the diffusion through the free edges needs to be evaluated for the composite, in addition to considering the anisotropic nature of the diffusion process.

**Figure 4:**
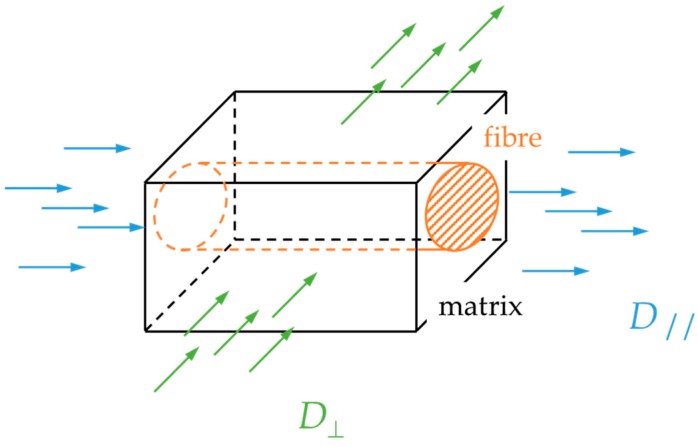
Simplified geometry of unidirectional composite for diffusion parallel and transverse to the fibers.

Starink, Starink and Chambers [20] have presented a modified treatment of diffusion in unidirectional composites assuming that the fibers do not take any moisture, where the diffusivity parallel (*D*_∥_) and transverse (*D*_⊥_) to the fibers in Figure 4 are given by

(10) $$D_{∥}=D_{r}$$

(11) $$D_{⊥}=\frac{\left(1-2\sqrt{\frac{v_{f}}{\pi }}\right)}{1-v_{f}}D_{r}=g^{2}\times D_{r}$$

where $v_{f}$ is the fiber volume fraction of the composite and $D_{r}$ is the diffusivity coefficient of the neat resin. The fiber hindrance factor *g*^2^ is an expression of the reduced free path for the diffusion species to follow due to the volume fraction taken by the inert inorganic fibers. It does not take into account any possible contribution due to the possible different chemical activity of the fiber-matrix interphase.

**Figure 5:**
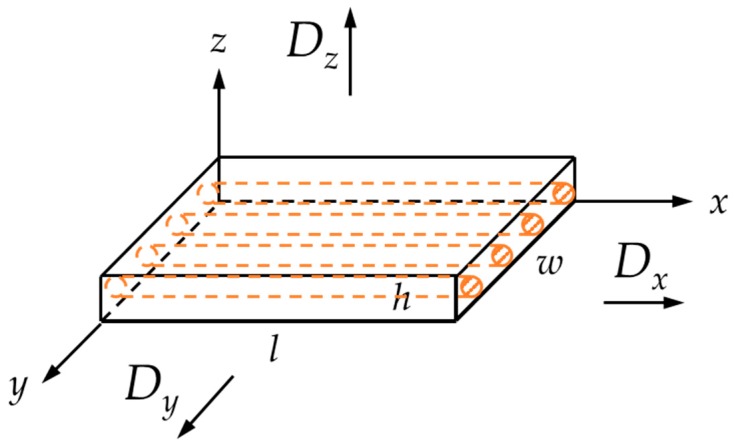
Schematic of rectanguloid anisotropic material specimen.

For the unidirectional composite in Figure 5, the diffusivity coefficients are

(12) $$D_{x}=D_{∥},\;D_{y}=D_{z}=D_{⊥}$$

Similar to the isotropic case, Starink, Starink and Chambers [20] have proposed a correction factor for an orthotropic medium, ignoring second order edge effects:

(13) $$f_{ortho}=1+0.54\frac{h}{w}\sqrt{\frac{D_{y}}{D_{z}}}+0.54\frac{h}{l}\sqrt{\frac{D_{x}}{D_{z}}}$$

By combining Equation (13) and Equations (10)–(12), it can be found that for the unidirectional composite, the correction factor is only a function of the geometry of the specimen, considering along which main direction the diffusion is favored (usually the shortest) and the fiber volume fraction. For the fibers aligned along the *x*-axis, as in Figure 5, this results in two possible scenarios:

(14a) $$\begin{array}{c} f_{ortho,\;⊥}=1+0.54\left(\frac{h}{w}+\frac{h}{l}\frac{1}{g}\right)\\ \mathrm{for}\;\mathrm{diffusion}\;\mathrm{mainly}\;⊥\;\mathrm{to}\;\mathrm{the}\;\mathrm{fibers} \end{array}$$

(14b) $$\begin{array}{c} f_{ortho,\;∥}=1+0.54\left(\frac{h}{w}+\frac{h}{l}\right)g\\ \mathrm{for}\;\mathrm{diffusion}\;\mathrm{mainly}\;∥\;\mathrm{to}\;\mathrm{the}\;\mathrm{fibers} \end{array}$$

Therefore, the effective diffusivity for the composite transverse and perpendicular to the fiber direction can be estimated as

(15a) $$D_{eff.\;⊥}≅f_{ortho,\;⊥}^{2}D_{⊥}=f_{ortho,\;⊥}^{2}\cdot g^{2}\cdot D_{r}$$

(15b) $$D_{eff.\;∥}≅f_{ortho,\;∥}^{2}D_{∥}=f_{ortho,\;∥}^{2}\cdot D_{r}$$

**Figure 6:**
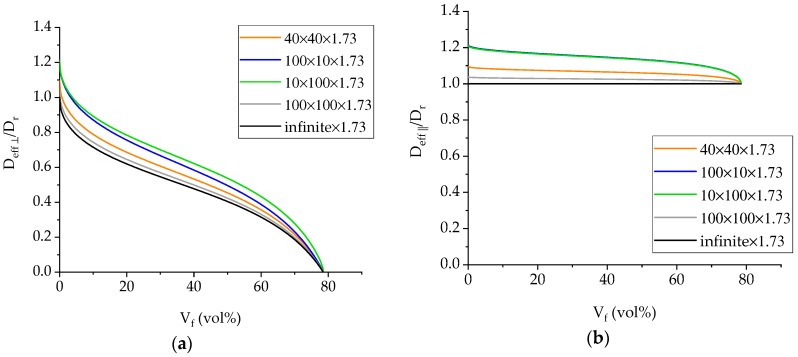
(**a**) *D_eff,_*_⊥_/*D_r_* and (**b**) *D_eff_*_,∥_/*D_r_* versus fiber volume fraction for the different shape specimens used in the study.

The above relationships are plotted in Figure 6 with respect to the volume fraction of the composite material. It is apparent that the 100 × 100 specimen geometry is closer to the infinite plate solution than any other geometry, hence providing the most accurate geometry for the measurement of the diffusivity.

The GFRP composite under investigation was found to have a fiber volume fraction of 56%. For this $V_{f}$ value, *g*^2^ is equal to 0.3536. These values were used in the calculation of the correction factors.

In Table 6, the correction factors and diffusivity coefficients for the composite material are listed. The value of the diffusivity calculated from the experimental weight gain test curves following the Fickian approach is denoted as *D_c_*. The corrected values *D_c,S&S_* and *D_c,SSC_* were calculated from Equation (7) combined with Equation (8) and Equation (14a), respectively. In addition, the effective diffusivity transverse (⊥) and parallel (∥) to the fibers were calculated from Equation (15a–b) and presented for comparison. The corrected diffusivity of the neat Ampreg 26 was used in the prediction of the effective anisotropic diffusivity.

In all cases, the least correction is required for the gamma type coupons, confirming the convergence to the infinite specimen size. In analogy with the neat Ampreg 26 case, it is evident that the correction proposed by Shen and Springer [19] seems to underestimate the material’s diffusivity (i.e., highest values of the correction factor), while the one proposed in [20] is more consistent and converges the diffusivity values towards the experimental of the 100 × 100 gamma type coupon, which is the representative closest to the ideal one-dimensional diffusion case.

**Figure 7:**
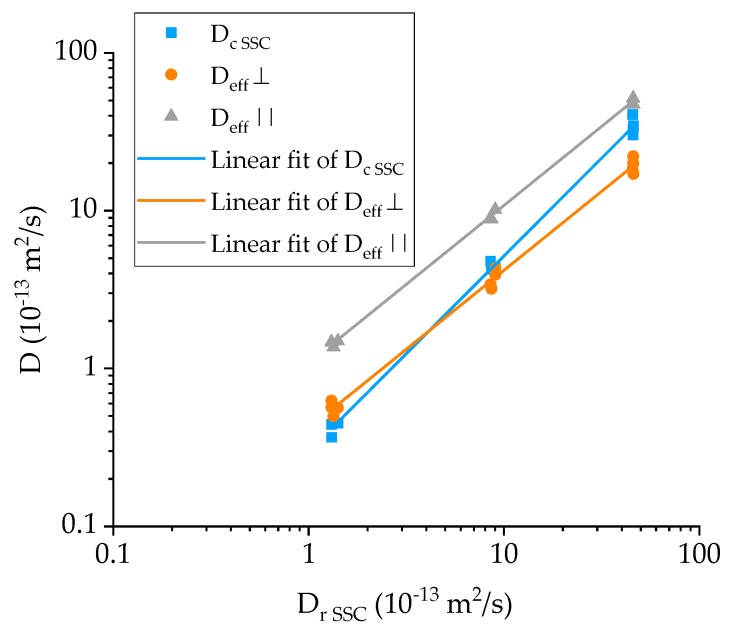
Plot of the effective and measured diffusivity coefficient for the GFRP composites against that of the corrected epoxy matrix. Note that all temperatures and specimen sizes were considered.

The results in Table 6, also plotted in Figure 7 with respect to the diffusivity of the resin, suggest that:

1. The effective diffusivity transverse to the fiber direction (*D_eff_*_,⊥_) is approximately half than of the resin’s, while the diffusivity along the fibers (*D_eff_*_,∥_) is slightly higher than that of the resin;
2. There is satisfactory agreement for the values of the GFRP diffusivity at 25 °C to *D_eff_*_,⊥_ but it progressively deviates at higher temperatures towards *D_eff_*_,∥_. This is an indication that while at ambient temperature, diffusion is occurring mainly through the thickness of the specimen, and as the temperature increases, there is significantly more seawater travelling through the edges of the specimens and along the fibers, which in turn suggests possible weakening of the fiber-matrix interface, providing an easy pathway for the diffusing liquid.

### 4.3. Arrhenius Plot and Prediction of Low Temperature Diffusivity

**Figure 8:**
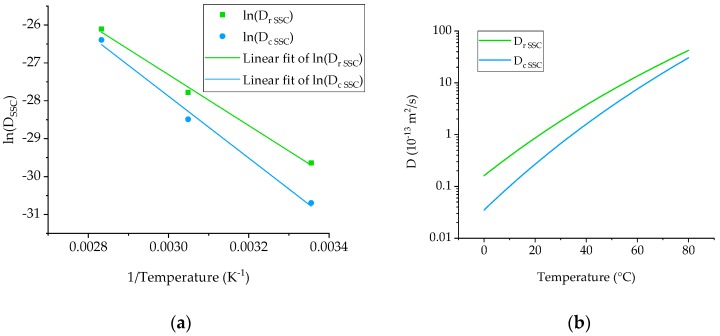
(**a**) Arrhenius plot and (**b**) predicted diffusivities for the neat Ampreg 26 and GFRP materials. Note that gamma specimens (quasi-infinite) are considered only.

It has been suggested [22,23] that the temperature dependence of diffusivity follows the Arrhenius equation as

(16) $$D\left(T\right)=Ae^{\frac{-E_{a}}{RT}}$$

where *A* is a pre-exponential factor and a constant for each chemical reaction, *T* is the absolute temperature in Kelvin, *E_a_* the activation energy for the reaction, and *R* is the universal gas constant equal to 3.13446 J/mol·K. To explore this assumption for the materials under investigation, an Arrhenius plot (*lnD* vs. (1/*T*)) was constructed for the gamma type specimen (Figure 8a). Despite the higher exposure temperature being very close to the glass transition temperature of the dry materials, it appears that the Arrhenius relationship holds for the range of temperatures investigated and an extrapolation can be safely made to lower temperatures (Figure 8b). Considering the calculated activation energies of 54.6 kJ/mol and 66.4 kJ/mol for the neat Ampreg 26 and the GFRP, respectively, the diffusivity at a temperature of 4 °C, representing realistic deep-water applications, was evaluated and shown to be equal to 0.23 and 0.05 × 10^−13^ m^2^/s.

### 4.4. Tg Measurements on Neat Ampreg 26 and GFRP Composite

**Figure 9:**
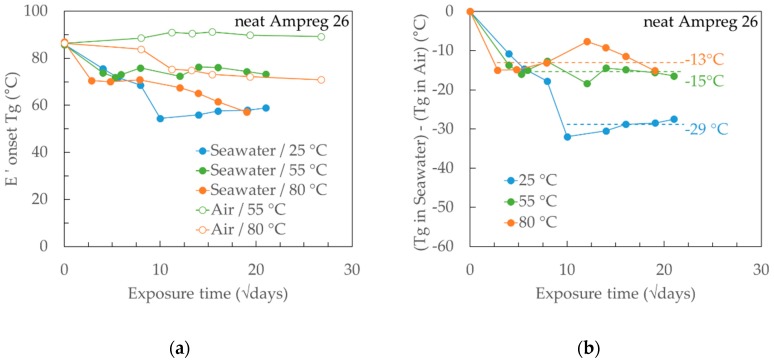
(**a**) Measured *Tg* (*E*′ onset) for A26 epoxy in seawater and dry air at different temperatures and (**b**) calculated *Tg* difference between wet and dry exposures for the A26 epoxy. The x-axes are measured in the square root of days.

**Figure 10:**
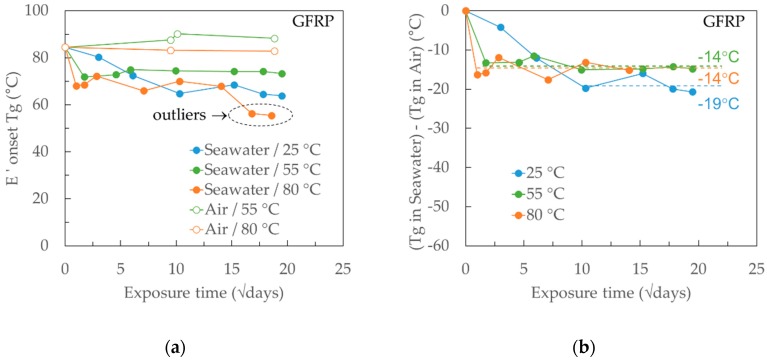
(**a**) Measured *Tg* (*E*′ onset) for GFRP in seawater and dry air at different temperatures and (**b**) calculated *Tg* difference between wet and dry exposures for the GFRP. The x-axes are measured in the square root of days.

In parallel to the gravimetric measurements, the change in the glass transition temperature (*Tg*) was monitored through an extensive campaign of Dynamic Mechanical Analysis (DMA) tests on progressively exposed specimens in seawater and dry air at different temperatures. The *Tg* was taken from the onset of the drop in the storage modulus (*E*′) in the DMA curve. The results are presented in Figure 9a and Figure 10a for the neat Ampreg 26 and GFRP composite, respectively. It can be noticed that for any curve referring to *Tg* after seawater exposure, the first local minimum happens at about the same time as the saturation of the material at the respective temperature when compared to the gravimetric curves, and the higher the exposure temperature, the faster this initial drop in the *Tg*.

The materials show a complex behaviour, but some common features can be highlighted. For the epoxy resin (see Figure 9a), the 25 °C seawater exposure shows a monotonic trend in the *Tg*, which is seemingly proportional to the seawater uptake, as recorded in the gravimetric test. The higher temperature exposures, instead, have alternated shifts. The measured *Tg* following exposure in dry air at various elevated temperatures did not reveal any additional chemical crosslink happening to the material, manifested through a rise in *Tg*, verifying that the epoxy system is properly cured at the start of the exposure tests [24].

To investigate the potential effect of the elevated exposure temperature, the *Tg* measured from the specimens exposed in air was subtracted from that measured from the specimens in seawater. For 25 °C, the dry *Tg* value of 86 °C was considered as constant with exposure time. The resulting curves are shown in Figure 9b for the neat Ampreg *26*. If the values beyond saturation of the material and up to the full duration of the exposure (i.e., one year) are considered, it appears that the net seawater effect is relatively similar for the elevated temperatures and significantly greater for the 25 °C exposure.

**Figure 11:**
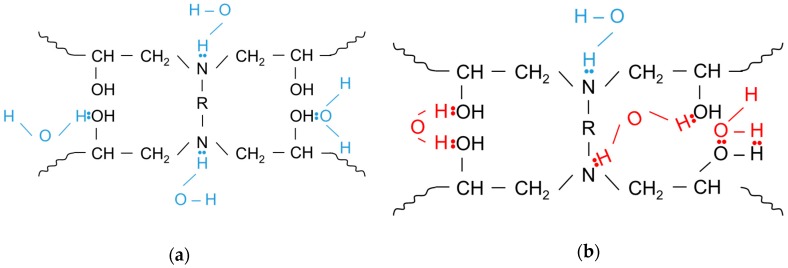
(**a**) Water molecules form one hydrogen bond with the epoxy network and (**b**) water molecules form more than one hydrogen bond with the epoxy network [25].

Zhou and Lucas [25] have articulated that there are two types of water bound in an epoxy’s crosslinked network. Type I bound water breaks inter-chain Van der Waals bonding and forms hydrogen bonds (Figure 11a). The net effect of Type I bound water is the increase in chain mobility that contributes to *Tg* depression. If only Type I bound water existed in the material, the *Tg* of the resin system would be independent of hygro-thermal exposure conditions; in other words, unaffected by exposure temperature and exposure time duration. In addition, there is Type II bound water that promotes secondary crosslinking with hydrophilic groups, such as hydroxyls and amines (Figure 11b). Popineau et al. [26] also postulated the existence of different types of water diffusing in epoxy, which are in agreement with the Langmuir-like diffusion kinetics proposed by Carter and Kibler [27]. However, they attributed different interactions of the water molecules to the polymer matrix, referring to them as free or bound solvent. There is no universal agreement about the behaviour of water inside a polymeric medium beside the initial plasticization effect [28]. Yet, different authors have proposed the dual type mechanism to explain the evolution of glass transition shifts [29,30,31].

Zhou and Lucas proved that the amount of Type II bound water increases with immersion time and a higher immersion temperature [25]. Therefore, the measured *Tg* value is influenced by a dual-mechanism process. That is, Type I bound water causes a drop in *Tg* and Type II bound water lessens the drop in *Tg* because of secondary crosslinking as a result of the water-thermoset network interaction.

This approach can explain the *Tg* decrease trend depicted in Figure 9b, where the exposure at 25 °C has caused much greater depression (Δ*Tg* = −29 °C) when compared to the exposures at 55 °C and 80 °C (Δ*Tg* of −15 °C and −13 °C, respectively), where the amount of Type II bound seawater would be much greater. When the absorbed seawater was removed by drying the specimens at 50 °C, there was a full recovery of the *Tg* with measured values of 87 °C, 91 °C, and 88 °C when the initially dry *Tg* of the material was measured in between 85 and 88 °C. It has been suggested that the recovery of the *Tg* is associated with the removal of Type I bound seawater that restores inter-chain Van der Waals bonding, which in turn masks the secondary cross-linking effect on *Tg*.

Similar to the epoxy, the variation in *Tg* of the GFRP composite with exposure time is presented in Figure 10a, while the net seawater effect, expressed as the difference between the *Tg* after seawater exposure and that after dry air exposure at the same temperature, is given in Figure 10b. The measured *Tg* shows the same monotonic trend, seemingly proportional to the seawater uptake, as recorded in the gravimetric tests. As for the neat epoxy, the depression in *Tg* was very close, irrespective of the elevated exposure temperatures, particularly if the last two points in the curve representing the 80 °C are treated as outliers since their respective *Tg* depression could not be replicated when the tanδ *Tg* value was considered. There was a greater *Tg* decrease for the 80 °C exposure, although the difference to the elevated temperatures was not as high. It is believed that in addition to the two mechanisms described earlier affecting the *Tg* depression, there is a third one related to the presence of the glass fibers and in particular, the interaction of the fiber-matrix interface with the absorbed seawater. Again, when specimens were dried following one-year exposure to seawater, the *Tg* was fully recovered with measured values of 84 °C, 89 °C, and 84 °C, when the initially dry *Tg* of the material was measured in between 81 and 88 °C.

If the *Tg* decrease was only influenced by the polymer plasticization and enhanced inter-chain mobility, then the Gordon-Taylor model [32] (see Equation (17)) would describe the relationship to the seawater content and all measured *Tg* values would lie along the same curve, irrespective of the exposure temperature. Instead, the parameter *k* in the model is different for different exposure temperatures, denoting a significant effect of temperature and reinforcing the assumption of different mechanisms affecting *Tg*.

**Figure 12:**
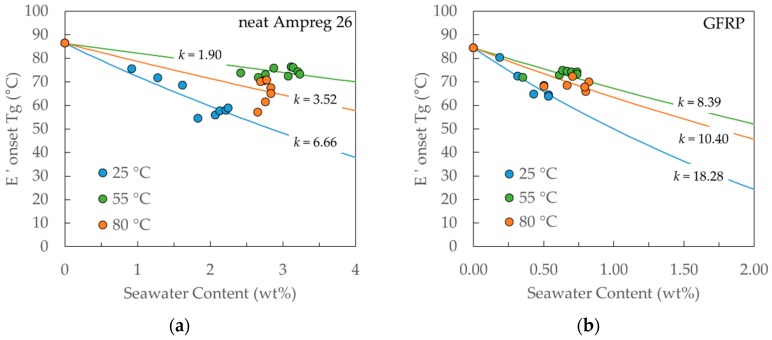
Correlation between the experimental results for (**a**) neat Ampreg 26 and (**b**) GFRP composite and Gordon-Taylor model, for *Tg* evolution with seawater content.

The Gordon-Taylor model describes the glass transition temperature *Tg* of a polymer as

(17) $$Tg=\frac{\left(1-x_{w}\right)Tg_{dry}+kx_{w}Tg_{w}}{\left(1-x_{w}\right)+kx_{w}}$$

where *x_w_* is the seawater content, *Tg_w_* is the glass transition temperature of the seawater taken to be equal to that of water (−137 °C) [32], *Tg_dry_* is the glass transition temperature of the dry material, and *k* is a material parameter denoting the effect of the presence of seawater beyond the linear rule of mixtures (i.e., *k* = 1). From Figure 12, it is suggested that the effect of seawater content is greater for the GFRP composite, where a smaller amount of absorbed liquid causes a greater *Tg* decrease and hence *k*_GFRP_ > *k*_resin_ for all exposure temperatures.

## 5. Conclusions

An extensive ageing campaign on epoxy and epoxy-based GFRP materials was conducted. The aim was to investigate the evolution of the material properties when exposed to a simulated marine environment. Three different temperatures were chosen (25, 55, and 80 °C), to compare the different rates at which the material properties would change, due to the increased physical/chemical kinetics.

The absorption progress was mainly monitored by gravimetric measurements. The results show that the behaviour is not completely Fickian. In particular, for the GFRP composite, the anisotropic nature of the material influences the diffusion kinetics with increasing temperature, both due to the fiber presence and the changing conditions of the fiber-matrix interphase. Two different correction factors were used, with that proposed by Starink et al. [20] providing more accurate results. Nevertheless, it is critical to use coupons with a width/length to thickness ratio of 50:1 or more (quasi-infinite plate) to reduce the effect of the fluid diffusion through the edges.

Using an Arrhenius plot, the exponential relation between the diffusivity coefficient and the exposure temperature was verified and the diffusivity coefficient values for the materials at a temperature of 4 °C, typical of offshore operative scenarios, were estimated at 0.23 and 0.05 × 10^−13^ m^2^/s for the neat Ampreg 26 and the composite, respectively.

In addition to the gravimetric measurements, tensile tests were performed on both materials to evaluate the evolution of mechanical performance with exposure to seawater. Although the experimental results are not presented in this paper, a close correlation between the performance loss, the degree of saturation, and the exposure temperature was found.

The shifts in the glass transition temperature for the different exposure conditions show that the initial local minimum happens at about the same time as the material saturates. The data was analysed in light of the findings reported by Zhou and Lucas [25] about the two types of bonding of water molecules with the epoxy network. The different bonding can increase chain mobility or induce secondary cross-linking, hence altering the materials’ *Tg*. Moreover, for the GFRP composite, the fiber-matrix interphase seems to play a role in the plasticization effect due to water absorption. When the specimens are re-dried after exposure, the recovery of *Tg* is almost complete, so the process seems to be mostly reversible. From an analysis of the data using the Gordon-Taylor model, it is evident that the plasticization effect is not just dependent on the water content, but also the temperature, and the different type of chemical bonds promoted do influence it.

Finally, the results verify the feasibility of accelerating the diffusion of seawater in polymer and polymer composites as a reliable way to recover the diffusivity factors at different temperatures and to estimate them for temperatures well below the glass transition of the polymer. The evaluation of the evolution of the glass transition temperature (*Tg*) is more complex due to the concurring mechanism interacting with the polymer network.

## Figures and Tables

**Table 1:** Standard substitute seawater as per ASTM D1141 [15].

Compound	Concentration
	(g/L)
NaCl	24.53
MgCl_2_	5.20
Na_2_SO_4_	4.09
CaCl_2_	1.16
KCl	0.695
NaHCO_3_	0.201
KBr	0.101
H_3_BO_3_	0.027
SrCl_2_	0.025
NaF	0.003
Other metal nitrates/nitrites	<0.1 mg/L
H_2_O	balance

**Table 2:** Diffusion test coupons’ nominal geometries for both neat Ampreg 26 and GFRP.

Code	*h* × *w* × *l*
	(mm × mm × mm)
α	2 × 19 × 19
β	2 × 40 × 40
γ—quasi-infinite	2 × 100 × 100
δ ^1^—fibers along l direction	2 × 10 × 100
ε ^1^—fibers along w direction	2 × 100 × 10

^1^ there is no physical difference between δ and ε for the neat Ampreg 26 (there is no fibre reinforcement); the 10 × 100 samples were unequivocally named as δ.

**Table 3:** Saturation levels and times for neat Ampreg 26 at various temperatures and specimen shapes. (Note: Mean values of three specimens for each geometry are listed).

Temperature	Specimen	*h* × *w* × *l*	Slope	*M*∞	*t*∞
(°C)		(mm × mm × mm)	$\left(\times \;10^{-4}\;\%/\sqrt{s}\right)$	(%)	(days)
25	alpha	2.09 × 18.86 × 19.15	8.8	1.94	113
	beta	2.18 × 40.00 × 40.08	7.9	1.90	107
	gamma	2.04 × 100.04 × 100.07	8.4	2.01	110
	delta	2.15 × 10.08 × 100.03	8.2	1.91	107
55	alpha	2.11 × 18.96 × 19.05	28.4	2.69	55
	beta	2.16 × 40.03 × 39.99	26.6	2.60	33
	gamma	2.12 × 100.05 × 100.05	26.6	2.63	33
	delta	2.07 × 10.06 × 100.05	28.4	2.91	48
80	alpha	2.15 × 19.31 × 19.14	71.7	2.77	16
	beta	2.15 × 39.96 × 40.03	65.9	2.77	9
	gamma	2.13 × 100.03 × 100.05	64.9	2.79	9
	delta	2.12 × 10.05 × 99.97	69.9	2.73	9

**Table 4:** Saturation levels and times for GFRP at various temperatures and specimen shapes. (Note: Mean values of three specimens for each geometry are listed).

Temperature	Specimen	*h* × *w* × *l*	Slope	*M*∞	*t*∞
(°C)		(mm × mm × mm)	$\left(\times \;10^{-4}\;\%/\sqrt{s}\right)$	(%)	(days)
25	beta	1.72 × 39.99 × 40.00	1.4	0.48	143
	gamma	1.70 × 99.84 × 99.73	1.3	0.46	132
	delta	1.72 × 9.99 × 99.96	1.4	0.51	182
	epsilon	1.70 × 99.90 × 10.03	1.5	0.47	119
55	beta	1.73 × 40.00 × 40.04	6.2	0.65	29
	gamma	1.70 × 99.91 × 99.98	5.9	0.67	41
	delta	1.73 × 9.95 × 99.98	6.1	0.64	31
	epsilon	1.72 × 99.93 × 9.99	6.6	0.66	29
80	beta	1.73 × 39.99 × 39.99	20.0	0.72	8
	gamma	1.76 × 99.12 × 99.75	20.0	0.82	24
	delta	1.71 × 10.00 × 100.03	19.6	0.75	17
	epsilon	1.74 × 99.93 × 10.01	21.1	0.80	70

**Table 5:** Diffusivity coefficient for neat Ampreg 26 at various temperatures and specimen shapes. (Note: Mean values of three specimens for each geometry are listed).

Temperature	Specimen	*h* × *w* × *l*	*f_S_*_&*S*_	*f_SSC_*	*D_r_*	*D_r,S_*_&*S*_	*D_r,SSC_*
(°C)		(mm × mm × mm)			(× 10^−13^ m^2^/s)		
25	alpha	2.09 × 18.86 × 19.15	1.22	1.12	1.74	1.17	1.38
	beta	2.18 × 40.00 × 40.08	1.11	1.06	1.59	1.29	1.41
	gamma	2.04 × 100.04 × 100.07	1.04	1.02	1.40	1.29	1.34
	delta	2.15 × 10.08 × 100.03	1.24	1.13	1.67	1.09	1.31
55	alpha	2.09 × 18.86 × 19.15	1.22	1.12	9.57	6.43	7.59
	beta	2.16 × 40.03 × 39.99	1.11	1.06	9.57	7.80	8.53
	gamma	2.12 × 100.05 × 100.05	1.04	1.02	9.03	8.31	8.63
	delta	2.07 × 10.06 × 100.05	1.23	1.12	8.02	5.33	9.01
80	alpha	2.15 × 19.31 × 19.14	1.22	1.13	61.09	40.80	48.27
	beta	2.15 × 39.96 × 40.03	1.11	1.06	51.01	41.60	45.49
	gamma	2.13 × 100.03 × 100.05	1.04	1.02	48.13	44.28	45.98
	delta	2.12 × 10.05 × 99.97	1.23	1.13	58.21	38.32	45.83

**Table 6:** Diffusivity coefficient for CFRP at various temperatures and specimen shapes. (Note: Mean values of three specimens for each geometry are listed).

Temp.	Specimen	*h* × *w* × *l*	*f_S_*_&*S*_	*f_ortho,_*_⊥_	*f_ortho,_*_∥_	*D_c_*	*D_c,S_*_&*S*_	*D_c,SSC_*	*D_eff,_*_⊥_	*D_eff,_*_∥_
(°C)		(mm × mm × mm)				(× 10^−13^ m^2^/s)				
25	beta	1.72 × 39.99 × 40.00	1.09	1.06	1.03	0.51	0.43	0.45	0.56	1.49
	gamma	1.70 × 99.84 × 99.73	1.03	1.02	1.01	0.49	0.46	0.47	0.50	1.37
	delta	1.72 × 9.99 × 99.96	1.19	1.11	1.06	0.45	0.31	0.37	0.57	1.47
	epsilon	1.70 × 99.90 × 10.03	1.19	1.16	1.06	0.60	0.43	0.44	0.63	1.47
55	beta	1.73 × 40.00 × 40.04	1.09	1.06	1.03	5.40	4.58	4.78	3.40	9.01
	gamma	1.70 × 99.91 × 99.98	1.03	1.02	1.01	4.46	4.17	4.25	3.20	8.82
	delta	1.73 × 9.95 × 99.98	1.19	1.11	1.06	5.37	3.78	4.36	3.92	10.15
	epsilon	1.72 × 99.93 × 9.99	1.19	1.17	1.06	5.75	4.06	4.23	4.33	10.14
80	beta	1.73 × 39.99 × 39.99	1.09	1.06	1.03	45.81	38.80	40.57	18.17	48.06
	gamma	1.76 × 99.12 × 99.75	1.04	1.03	1.01	36.29	33.85	34.50	17.10	47.03
	delta	1.71 × 10.00 × 100.03	1.19	1.11	1.06	39.91	28.27	32.52	19.89	51.53
	epsilon	1.74 × 99.93 × 10.01	1.19	1.17	1.06	40.94	28.84	30.05	22.08	51.63

## Appendix A

**Table A1:** Comparison of the results obtained at effective equilibrium for the gravimetric test of the neat Ampreg 26 using different equilibrium criteria. (Note: mean values of three coupons for each geometry are listed).

Effective Equilibrium Criterion	Temperature		25 °C				55 °C				80 °C			
	Specimen		alpha	beta	gamma	delta	alpha	beta	gamma	delta	alpha	beta	gamma	delta
ASTM D5229M Equation (1)	Effective equilibrium ^1^	(days)	131	145	117	113	70	63	76	55	40	22	35	9
	Moisture content	(%)	1.93	1.96	1.99	1.88	2.66	2.63	2.68	2.85	2.78	2.78	2.79	2.75
	Uptake rate ^2^	(10^−8^/s)	1.48	0.65	2.88	1.59	1.29	2.57	1.70	3.00	0.90	0.35	8.60	3.30
Criterion A as Equation (2) (0.02, *n* = 2)	Effective equilibrium	(days)	113	107	110	107	55	33	33	48	16	9	9	9
	Moisture content	(%)	1.94	1.90	2.01	1.91	2.69	2.60	2.63	2.91	2.77	2.77	2.79	2.73
	Uptake rate	(10^−8^/s)	0.16	2.00	2.90	3.00	1.70	1.67	2.95	1.12	1.60	2.60	2.60	1.20
Criterion B as Equation (2) (0.05, *n* = 3)	Effective equilibrium	(days)	62	62	65	62	27	26	28	35	8	8	9	8
	Moisture content	(%)	1.63	1.59	1.75	1.62	2.52	2.49	2.57	2.76	2.69	2.73	2.75	2.75
	Uptake rate	(10^−8^/s)	4.87	6.56	8.01	6.34	4.90	5.12	6.40	6.00	4.80	1.00	2.60	1.10

^1^ The time to effective equilibrium is rounded up to the next integer. ^2^ The uptake rate is the average time ratio of the percent weight gain; in other words, a saturation “speed”.
